# Predictability of B cell clonal persistence and immunosurveillance in breast cancer

**DOI:** 10.1038/s41590-024-01821-0

**Published:** 2024-05-02

**Authors:** Stephen-John Sammut, Jacob D. Galson, Ralph Minter, Bo Sun, Suet-Feung Chin, Leticia De Mattos-Arruda, Donna K. Finch, Sebastian Schätzle, Jorge Dias, Oscar M. Rueda, Joan Seoane, Jane Osbourn, Carlos Caldas, Rachael J. M. Bashford-Rogers

**Affiliations:** 1 Breast Cancer Now Toby Robins Research Centre, The Institute of Cancer Research, London, UK; 2 The Royal Marsden Hospital NHS Foundation Trust, London, UK; 3 Alchemab Therapeutics, Whittlesford, UK; 4 Wellcome Centre for Human Genetics, Oxford, UK; 5 Nuffield Department of Clinical Neuroscience, University of Oxford, Oxford, UK; 6 Cancer Research UK Cambridge Institute, University of Cambridge, Cambridge, UK; 7 IrsiCaixa, Germans Trias i Pujol University Hospital, Badalona, Spain; 8 Germans Trias i Pujol Research Institute (IGTP), Badalona, Spain; 9 MRC Biostatistics Unit, University of Cambridge, Cambridge, UK; 10 Vall d’Hebron Institute of Oncology (VHIO), Vall d’Hebron University Hospital, Institució Catalana de Recerca i Estudis Avançats (ICREA), Universitat Autònoma de Barcelona (UAB), CIBERONC, Barcelona, Spain; 11 School of Clinical Medicine, University of Cambridge, Cambridge, UK; 12 Department of Biochemistry, University of Oxford, Oxford, UK; 13 Oxford Cancer Centre, Oxford, UK

**Keywords:** Immunoediting, Clonal selection, Immunological surveillance

## Abstract

B cells and T cells are important components of the adaptive immune system and mediate anticancer immunity. The T cell landscape in cancer is well characterized, but the contribution of B cells to anticancer immunosurveillance is less well explored. Here we show an integrative analysis of the B cell and T cell receptor repertoire from individuals with metastatic breast cancer and individuals with early breast cancer during neoadjuvant therapy. Using immune receptor, RNA and whole-exome sequencing, we show that both B cell and T cell responses seem to coevolve with the metastatic cancer genomes and mirror tumor mutational and neoantigen architecture. B cell clones associated with metastatic immunosurveillance and temporal persistence were more expanded and distinct from site-specific clones. B cell clonal immunosurveillance and temporal persistence are predictable from the clonal structure, with higher-centrality B cell antigen receptors more likely to be detected across multiple metastases or across time. This predictability was generalizable across other immune-mediated disorders. This work lays a foundation for prioritizing antibody sequences for therapeutic targeting in cancer.

## Abstract (web summary)

In this Resource paper, the authors integrate T cell antigen receptor, B cell antigen receptor and exome sequencing comparing early and metastatic breast cancer in humans, showing how the immune response and tumors coevolve.

## Main

The mechanisms by which tumors evade immune control are critical to developing better targeted immunotherapies. B and T cells play an important role in anticancer immunity^1,2^. However, while the T cell immune response to cancer and its therapeutic manipulation is well characterized, the B cell contribution to antitumor immunity remains less well studied.

B cells contribute to antitumor responses by binding tumor antigens via their B cell antigen receptor (BCR) and presenting these to follicular helper T cells, by antibody secretion and by cytokine signaling to other cells. Tumor-infiltrating B cells are associated with improved clinical outcomes^3–6^ and response to chemotherapy and immunotherapy^7,8^, and the persistence of plasma antitumor antibodies and tumor-associated tertiary lymphoid structures (TLSs) associate with improved survival^4,9^.

B and T cell clones selectively expand following antigen recognition by their BCR and T cell antigen receptor (TCR), respectively. These receptors are generated through DNA recombination and have the potential to recognize a vast array of antigens. On encountering antigen, B cells can be stimulated to proliferate and further diversify their BCR sequences via class switching and somatic hypermutation (SHM) resulting in high-affinity B cell responses^10^. Previous studies in breast cancer have shown significant heterogeneity in tumor-infiltrating B cell subpopulations, significant levels of SHM and clonal expansion, and local differentiation of infiltrated memory B cells^11,12^. Indeed, some studies have shown that tumor-infiltrating B cells can have antitumor BCR specificities, such as anti-HER2 autoantibodies in breast cancer^13,14^.

The immune system can monitor, recognize and destroy transformed cells or pathogens, a concept termed immunosurveillance^15^. Immunosurveillance is responsible for shaping the tumor molecular landscape and is key to the effectiveness of anticancer therapies. However, despite the potential impact of B cells in antitumor responses and patient survival, the nature of B cell immunosurveillance during systemic anticancer therapy and across metastatic sites in breast cancer is unknown.

Here, we perform a comprehensive analysis of breast cancer immunosurveillance in metastatic and early breast cancer. By integrating BCR, TCR, DNA and RNA-sequencing (RNA-seq) data from a multisite metastatic cohort, and during neoadjuvant therapy in an early disease cohort, we tracked and characterized clones that were temporally persistent throughout therapy and across metastatic sites (spatio-migratory mapping). Using this data, we aimed to uncover three key features of B cell clonal temporal persistence and immunosurveillance. Firstly, to determine whether the intra-tumoral B cell response across metastases is correlated with the tumor genomic landscape and T cell response, in keeping with the immunoediting hypothesis. Secondly, to determine the nature of B cell immunosurveillance between metastatic sites and throughout anticancer therapy. Lastly, we sought to identify what key B cell clonal features predict immunosurveillance and temporal persistence for future therapeutic exploration.

## Results

### Multi-platform metastatic tumor profiling

**Fig. 1:**
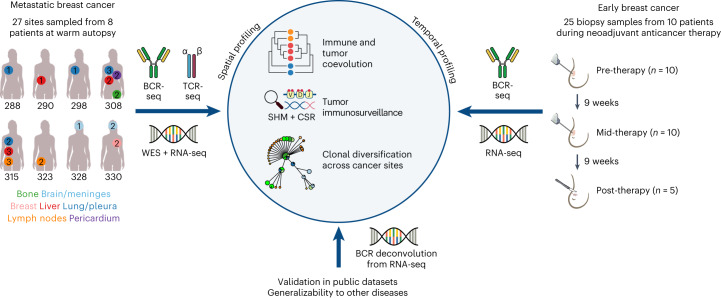
Description of breast cancer cohorts and overview of study design. Schematic of the sampling, data collection and analysis of the breast cancer cohorts in this study. Female silhouette is from the public domain diagrams of the human body at https://commons.wikimedia.org/wiki/Human_body_diagrams. WES, whole-exome sequencing.

**Extended Data Fig. 1:**
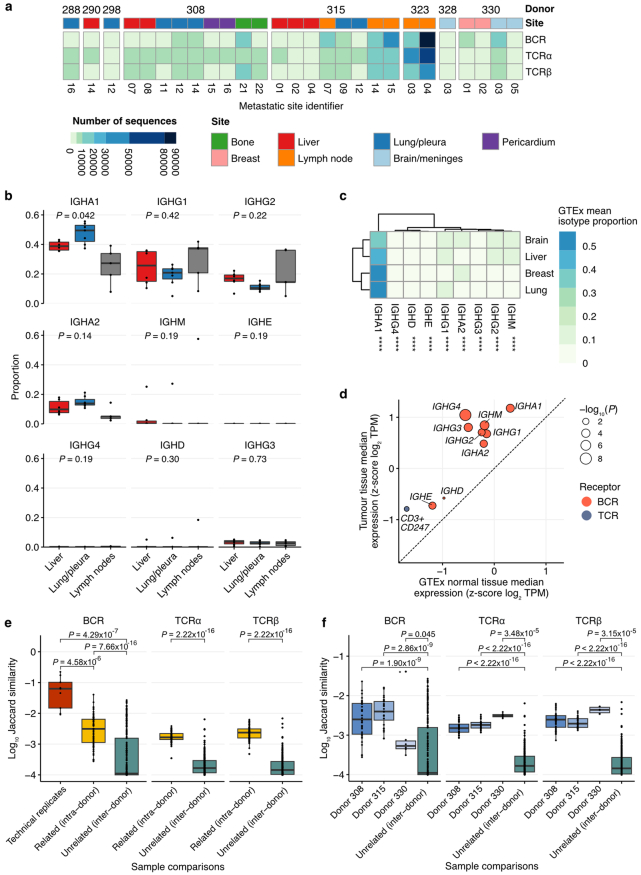
BCR sequencing quality-control statistics. **a**) Heatmap showing number of unique BCR and TCR sequences identified across 27 metastatic sites sampled from 8 patients. **b**) Box plots showing distribution of BCR isotype usages by metastatic site (n = liver: 6, lymph nodes: 5, lung/pleura: 7). *P* values were calculated using Kruskal-Wallis test and adjusted for multiple comparisons. **c**) Heatmap showing BCR IGH isotype usage across n = 3,905 healthy tissue samples in GTEx (****P < 6 × 10^−16^). *P* values were calculated using Kruskal-Wallis test and adjusted for multiple comparisons. **d**) Scatter plot showing expression of BCR isotypes and CD3/CD247 in tumour versus healthy tissues in GTEx. **e**) Box plots showing the log_10_ Jaccard BCR and TCR similarity between technical replicates (n = 9 comparisons), related samples derived from the same patient (n = 71 comparisons) and unrelated samples (n = 208 comparisons). **f**) Box plots showing the log_10_ Jaccard BCR and TCR similarity between samples obtained from patients 308 (n = 36 comparisons), 315 (n = 28 comparisons) and 330 (n = 6 comparisons), as well as unrelated samples (n = 208 comparisons). **d**–**f**) Wilcoxon rank sum tests, all *P* values two-sided. **b, e, f**) The box bounds the interquartile range divided by the median, with the whiskers extending to a maximum of 1.5 times the interquartile range beyond the box. Individual data points shown as dots.

We performed BCR repertoire sequencing on 27 metastatic tumor biopsy samples obtained through warm autopsies of eight participants with therapy-resistant metastatic breast cancer to identify B cell clonality, isotype usages and clonal diversification across the metastases (Fig. 1 and Supplementary Table 1). The mean yield of unique BCRs for each metastatic site after filtering was 9,332 (range, 701–80,409; Extended Data Fig. 1a and Methods). The genomic, transcriptomic and TCR repertoires of these metastatic tumors have been previously reported^16^.

**Fig. 2:**
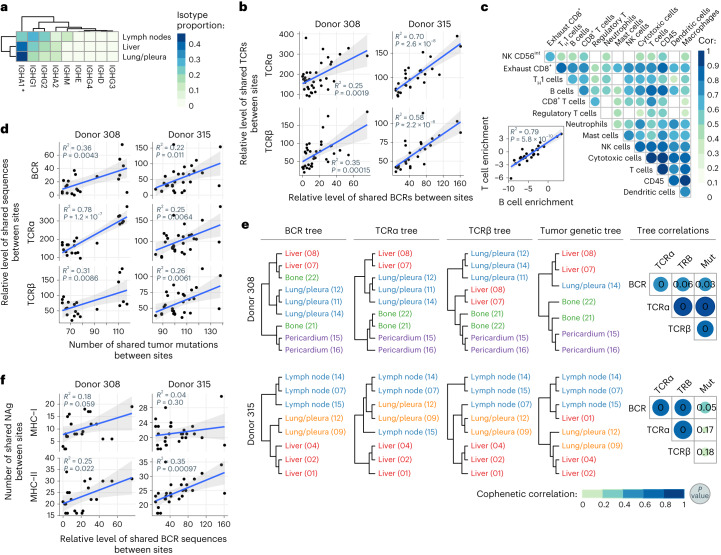
Site-specific B cell infiltration correlates with T cell infiltration and tumor genomic landscape. **a**, Mean BCR isotype usage across metastatic sites with more than two samples (lymph nodes *n* = 5, liver *n* = 6, lung/pleura *n* = 7; IGHA1 **P = 0.042). *P* values calculated using Kruskal–Wallis test and adjusted for multiple comparisons. **b**, Scatterplots showing the relative level of sharing of BCRs between sites against the relative level of sharing of TCRα/β VDJ sequences between pairwise metastatic site comparisons. **c**, Correlation between tumor immune microenvironment components deconvoluted from bulk RNA-seq data using Danaher gene sets. Inset, scatterplot showing relationship between T cell and B cell enrichment. *P* value and *R*^*2*^ obtained from linear regression. NK, natural killer cell, T_H_1, type 1 helper T cells. Data from all sites (*n* = 27) from all participants are shown. **d**, Scatterplots showing number of shared BCR and TCRα/β VDJ sequences and tumor mutations between pairwise metastatic site comparisons. **e**, Clonal similarity trees for BCR, TCRα/β VDJ sequences and mutational phylogenetic trees for participants 308 and 315. Inter-tree correlations shown on the left. One-sided *P* values derived from permutation tests shown within correlation circles. Trees have arbitrary units for branch lengths. **f**, Scatterplots showing number of shared BCR VDJ sequences and predicted MHC class I and II neoantigens (NAg) between pairwise metastatic site comparisons. **b**,**d**,**f**, BCR and TCR sequences were downsampled. *P* value and *R*^*2*^ obtained from linear regression analysis. The shaded area, in gray, represents the 95% confidence interval. Inter-sample comparisons: participant 308, *n* = 36; participant 315, *n* = 28.

Significant BCR isotype usage variations were observed across metastatic sites, with liver and lung/pleura dominated by IgA1 (Fig. 2a and Extended Data Fig. 1b). The distribution of BCR isotypes across metastatic sites was distinct from that observed in healthy normal tissues using deconvolution of bulk RNA-seq data from the Genotype-Tissue Expression (GTEx) Consortium atlas^17^ (Extended Data Fig. 1c and Methods). Additionally, there was a higher expression of both IGH and TCR genes in metastatic tumor tissues compared to normal tissues (Extended Data Fig. 1d). Together, these data suggest that the BCR and TCR patterns observed were the result of tumor-associated responses rather than reflecting healthy tissue heterogeneity.

### B cell and T cell clonal structures are correlated

B cell and T cell clones are defined by cells sharing related BCR or TCR VDJ rearrangements. We used the Jaccard index to quantify the degree of clonal sharing of the VDJ regions of the BCR, TCRα and TCRβ clones between sites (Extended Data Fig. 1e,f), revealing that BCR and TCR repertoires were distinct between each participant, in keeping with previous studies^18^. A low degree of BCR and TCR VDJ sequence sharing, which may occur by chance at low frequencies^19^, was observed between different participants, while high levels of BCR and TCR VDJ sharing were only observed in the metastases from the same participant.

**Extended Data Fig. 2:**
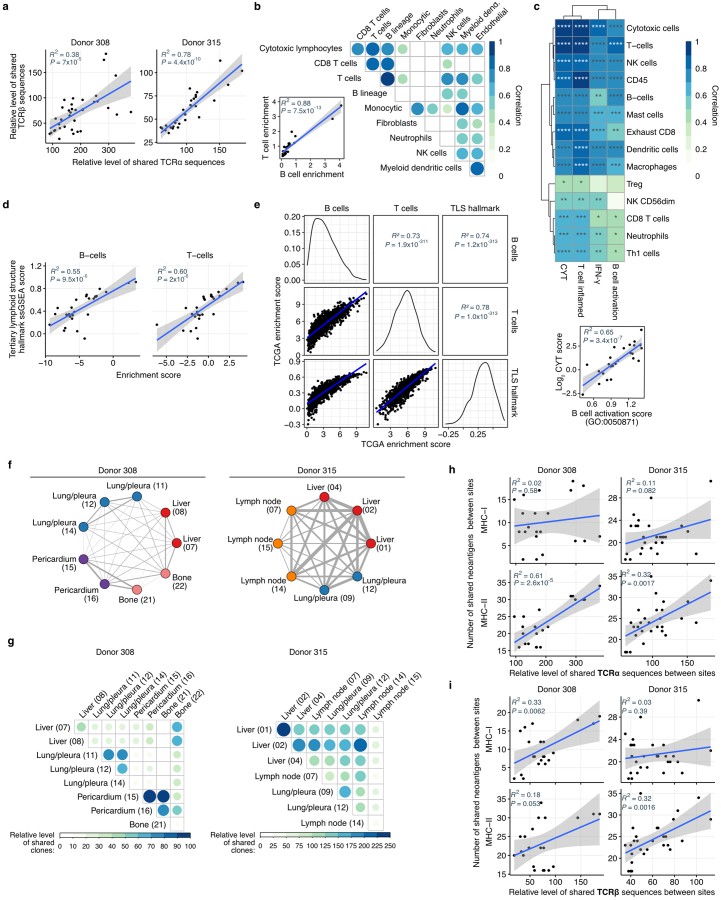
Adaptive immune and tumour co-evolution. **a**) Scatter plots showing relationship between shared TCRα and TCRβ VDJ sequences across sampled metastatic sites (inter-sample comparisons: patient 308 *n* = 36. patient 315: *n* = 28). TCR sequences down sampled. *P* value and *R*^*2*^ obtained from linear regression analysis. **b**) Correlation plot showing relationship between tumour immune microenvironment components deconvoluted from the bulk RNA-Seq data using MCPcounter. Inset: scatter plot showing relationship between T and B cell enrichment. *P* value and *R*^*2*^ obtained from linear regression. **c**) **Top:** heatmap showing Pearson’s correlations between tumour immune microenvironment composition (obtained using Danaher gene sets) and activity. Enrichment scores obtained using bulk RNA-Seq data. ****P < 0.0001, ***P < 0.001, **P < 0.01, *P < 0.05. **Bottom:** scatter plot showing correlation between B cell activation and cytolytic activity (CYT). *P* value and *R*^*2*^ obtained from linear regression analysis. **d**) Scatter plots showing relationship between B and T cell enrichment and expression of a tertiary lymphoid structure gene set. *P* value and *R*^*2*^ obtained from linear regression analysis. **e**) Relationship between B cell, T cell and tertiary lymphoid structure (TLS) hallmark signature in the TCGA breast cancer cohort (n = 1083 tumours). *P* value and *R*^*2*^ obtained from linear regression analysis. **f**) Spatio-migratory map of B cell clonal migration between metastatic sites. Edge width proportional to relative number of shared BCR clones between sites. **g**) Heatmaps showing relative number of shared BCR clones between sites. **h**) Scatter plots showing relationship between shared TCRα VDJ sequences and predicted MHC class I and II neoantigens across pairwise metastatic sites. **i**) Scatter plots showing relationship between shared TCRβ VDJ sequences and predicted MHC class I and II neoantigens across pairwise metastatic sites. **b**–**d**) Data from all sites (n = 27) from all patients used. **a**–**d**, **h**, **i**) The shaded area, in grey, represents the 95% confidence interval. **a**, **f-i**) Data from two patients with more than four metastatic sites sampled used (308, 315). **h**, **i**) TCR sequences were downsampled. *P* value and *R*^*2*^ obtained from linear regression analysis. Inter-sample comparisons: patient 308 *n* = 36; patient 315: *n* = 28.

We next compared the clonal structures across metastatic sites in the two participants in which BCR and TCR sequencing data were available for four or more sites (participants 308 and 315). TCRα and TCRβ clonal structures were correlated across metastases (strong correlation in participant 315 and, to a lesser degree, but also significant, in participant 308; Extended Data Fig. 2a), in keeping with the common origin of these receptors. BCR clonal structures across metastatic sites were also correlated with TCRα and TCRβ clonal structures, indicating shared factors driving B cell and T cell infiltration and selection (Fig. 2b). This was confirmed by deconvoluting tumor immune microenvironment composition and activity^20^ from the bulk RNA-seq data using the Danaher gene sets^21^ and MCPcounter^22^, which showed that both the abundance (*R*^*2*^ = 0.79, Fig. 2c and Extended Data Fig. 2b) and activation (*R*^*2*^ = 0.65; Extended Data Fig. 2c) of tumor-infiltrating B cells and T cells were strongly correlated. B cell and T cell enrichment was also significantly associated with the expression of a TLS signature (Extended Data Fig. 2d)^23^, in keeping with observations that coordination between BCR and TCR repertoires occurs within these structures^24^. This relationship was also observed in The Cancer Genome Atlas (TCGA) early breast cancer cohort (Extended Data Fig. 2e).

In summary, B cell and T cell infiltration, clonality and activation are significantly correlated across metastases, providing evidence that B cell and T cell responses are coordinated across metastatic sites in each individual breast cancer participant.

### Adaptive immune and tumor genomic coevolution

We previously showed that T cell responses, assessed by TCR sequencing, appear to coevolve with the metastatic tumor genomes^16^. This prompted us to investigate whether a similar association would be observed for the B cell response. In the two participants for which more than four metastases were sequenced, B cell and T cell clonal compositions mirrored the tumor mutational landscape, with significant associations observed between the number of shared TCRs, BCRs and somatic mutations across metastatic sites (*R*^*2*^ range, 0.22–0.78, *P* ≤ 0.011; Fig. 2d).

To confirm this, unsupervised VDJ BCR and TCR Jaccard phylogenetic trees segregated metastases by organ, with consistent clustering patterns between BCR, TCRα and TCRβ chains (Fig. 2e). Similar tree structures were observed when tumor mutational phylogenies were constructed from the whole-exome sequencing data (Fig. 2e). The BCR and TCR tree structures in both participants were significantly correlated when analyzed using the cophenetic statistic, with similar but weaker correlations observed when these were compared to the tumor mutational phylogenetic trees (Fig. 2e), providing further evidence that the tumor and the adaptive immune response coevolve. Finally, maps of B cell clonal structure across metastatic sites, generated through quantifying the degree of clonal sharing of the BCR clonotypes between sites (Extended Data Fig. 2f,g), confirmed that there was clonal overlap between most sites within an individual, but the levels were highly variable between sites.

We subsequently characterized correlations between predicted major histocompatibility complex (MHC) class I and II neoantigens and BCR and TCR clonal structure. There was a significant correlation between BCR clonal structure and shared MHC class II-predicted neoantigens (*R*^*2*^ range, 0.25–0.35, *P* < 0.022; Fig. 2f) but not MHC class I-predicted neoantigens. Similar observations were made with TCR clonal structure (Extended Data Fig. 2h,i), suggesting that B cell and T cell clonal structures significantly mirror tumor MHC class II-predicted neoantigen architecture.

In summary, each individual metastasis has a unique BCR and TCR clonal architecture. However, more similar BCR and TCR repertoires exist between metastases sharing similar mutational landscapes, suggesting coevolution between tumors and B cell and T cell responses across metastases.

### Persistence and immunosurveillance of intra-tumoral B cells

**Extended Data Fig. 3:**
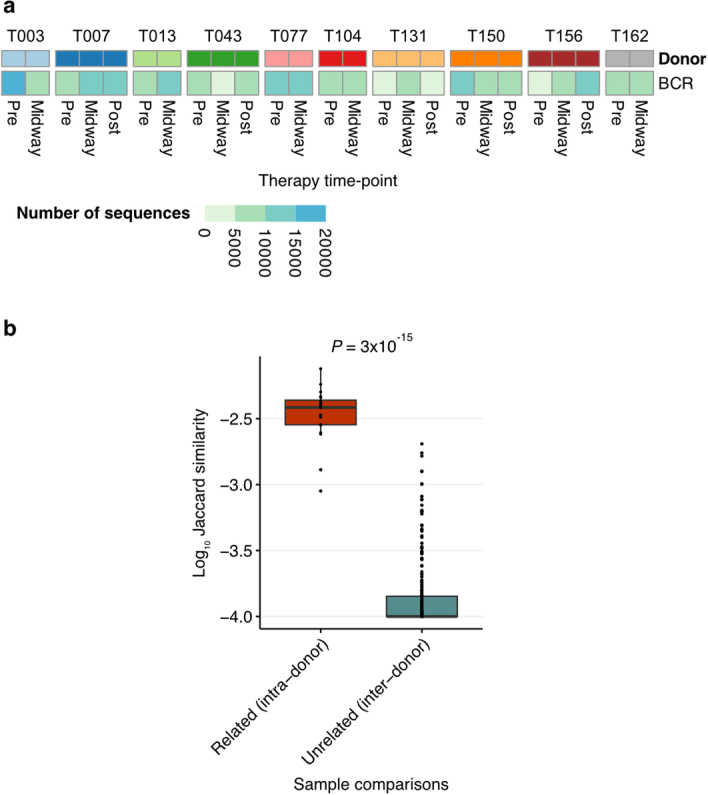
Early breast cancer cohort BCR sequencing metrics. **a**) Heatmap showing number of unique BCR sequences identified across 25 tumour biopsies sampled from 10 patients. **b**) Box plot showing the log_10_ Jaccard BCR similarity between related samples derived from the same patient (n = 20 comparisons) and unrelated samples (n = 280 comparisons). Wilcoxon rank sum test, *P* value two-sided. The box bounds the interquartile range divided by the median, with the whiskers extending to a maximum of 1.5 times the interquartile range beyond the box. Individual data points shown as dots.

We performed BCR repertoire sequencing on an early breast cancer cohort comprising ten participants with sequential tumor biopsy samples obtained during neoadjuvant therapy (25 serial samples: *n* = 10 before therapy, *n* = 10 after 9 weeks of therapy and *n* = 5 on completion of therapy). We obtained a mean yield of 8,132 unique BCRs per biopsy after filtering (range, 762–15,493; Extended Data Fig. 3a and Supplementary Table 2). Each participant harbored distinct BCR repertoires (Extended Data Fig. 3b). Together with the metastatic dataset, this allowed an exploration of the spatial and temporal nature of tumor-infiltrating B cells.

**Fig. 3:**
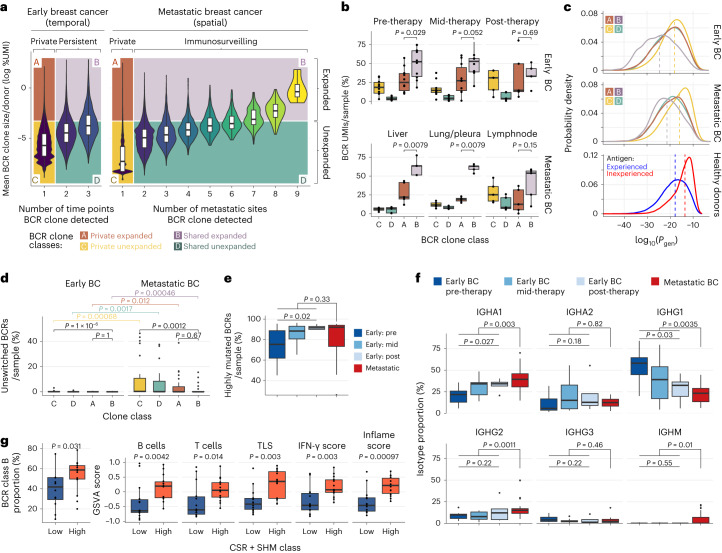
Immunosurveilling and persistent clones are enlarged and distinct from private clones. **a**, Violin plots of mean BCR clone sizes per sample across sampling time points in early breast cancer (*n* = 94,495 unique BCR clones) and across number of sites in metastatic breast cancer (*n* = 155,451 unique BCR clones). BCR clones classified as private expanded (class A, *n* = 10,507 clones), shared expanded (class B, *n* = 6,358 clones), private unexpanded (class C, *n* = 217,093 clones) and shared unexpanded (class D, *n* = 15,988 clones). **b**, Box plots showing percentage of expanded BCRs in early breast cancer (pre-therapy: *n* = 10, mid-therapy: *n* = 10, post-therapy: *n* = 5) and metastatic breast cancer (liver: *n* = 5, lymph node: *n* = 5, lung: *n* = 5) samples. **c**, Distribution of BCR CDR3 *P*_gen_ scores in the BCR clone classes in the early breast cancer and metastatic datasets. Distribution of BCR CDR3 *P*_gen_ scores in a comparative healthy peripheral blood mononuclear cell dataset across antigen-experienced (CSR and SHM) and antigen-inexperienced (no CSR and SHM) BCRs. **d**, Box plots showing the percentage of unswitched BCRs (IgD/IgM) per sample across the four BCR clone classes. **e**, Box plot showing the percentage of highly mutated BCRs in early and metastatic breast cancer samples. **f**, Box plots showing the percentage isotype usage in early and metastatic breast cancer samples. **g**, Box plots showing the distribution of the proportion of BCR class B clones, and signature scores of B cell, T cell, TLS, IFN-γ and T cell inflamed deconvoluted from bulk RNA-seq data between samples with low and high levels of SHM and CSR (high, >50th percentile (SHM, CSR); low, ≤50th percentile (SHM, CSR)). **a**,**c**,**d**,**g**, (Data (left) from participants with more than one tumor site sampled shown (early breast cancer cohort: all participants (*n* = 10), metastatic breast cancer cohort participants: 308, 315, 323 and 330). **e**,**f**,**g**, Data (right) from all participants (*n* = 18) and all samples (*n* = 52). **e**,**f**, *P* early breast cancer: ordinal regression, *P* early versus late breast cancer: Wilcoxon rank-sum tests. All *P* values are two sided. **b**,**d**,**e**,**g**, Wilcoxon rank-sum tests. All *P* values are two sided. **a**,**b**,**d**–**g**, The box bounds denote the interquartile range, the line indicates the median, and whiskers indicate maximum of 1.5 times the interquartile range beyond the box. Individual data points are shown as dots.

**Extended Data Fig. 4:**
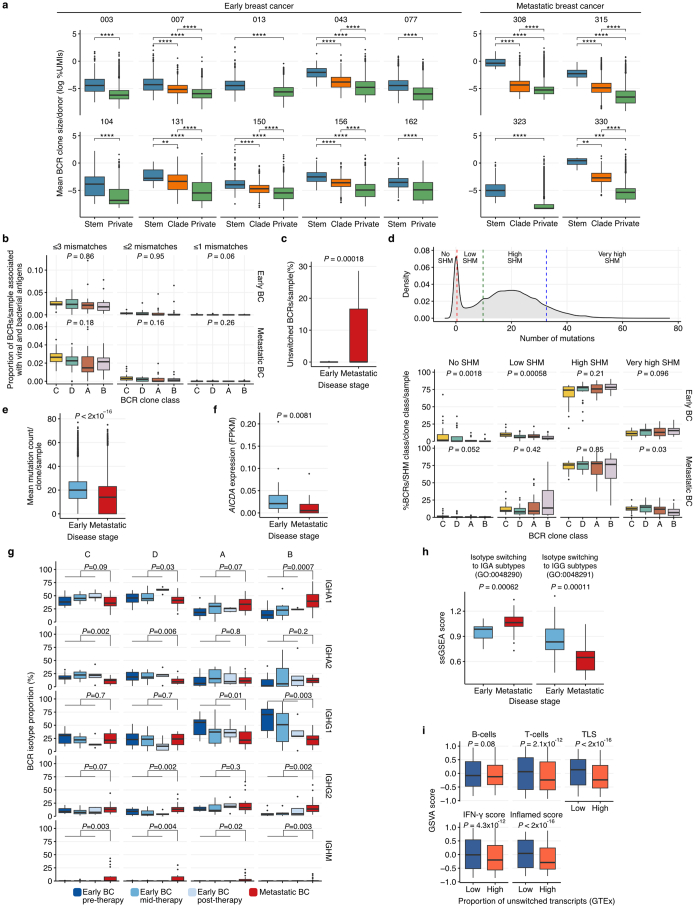
BCR clone classification analyses. **a**) Boxplots showing distribution of BCR clone sizes in stem, clade, and private BCR clones across early (n = 4487 stem, 4569 clade, 85439 private unique BCR clones) and metastatic cancer (n = 1268 stem, 12022 clade, 142161 private unique BCR clones) cohorts. ****P < 4.9 × 10^−5^, ***P = 0.00099, **P = 0.002, two-sided Wilcoxon Rank sum test and adjusted for multiple comparisons. **b**) Boxplots showing proportion of unique BCRs/sample with CDR3 sequences matching a reference antibody database with known binding to viral or bacterial antigens with ≤3, ≤2 and ≤1 CDR3 mismatches, across the four BCR clone classes. *P* values calculated using two-sided analysis of variance (ANOVA). **c**) Boxplot showing the percentage of unswitched BCRs per sample in early and metastatic breast cancer cohorts. *P* values calculated two-sided Wilcoxon Rank sum test. **d**) **Top:** density plot showing distribution of BCR SHM and thresholds used to classify BCRs in four SHM classes. **Bottom:** Boxplots showing the distribution of BCRs proportions in four SHM classes across the four BCR clone classes in early and metastatic breast cancer cohorts. *P* values calculated using Kruskal-Wallis test. **e**) Boxplot showing the mean mutation count per BCR clone per sample in early and metastatic breast cancer cohorts. **f**) Boxplot showing expression of *AICDA* in early and metastatic breast cancer cohorts. **g**) Boxplots showing % isotype usage in early and metastatic breast cancer samples across the four BCR clone classes. Wilcoxon rank sum tests, *P* values adjusted and two-sided. **h**) Boxplots showing distribution of enrichment scores of two RNA isotype switching signatures. **i**) Boxplots showing distribution of immune microenvironment cell type scores and activation signatures across n = 3,905 healthy tissue samples in GTEx. Samples with high expression of unswitched transcripts were defined as those with a >50th percentile expression of IGHD/M genes. **a**, **b**, **d**, **g**) Data from patients with more than one tumour site sampled shown (early breast cancer cohort: n = 10 patients, 25 samples; metastatic breast cancer cohort patients: 308, 315, 323, 330, n = 23 metastatic cancer samples) used. **c**,**e**,**f**,**h**) Data from all patients used (early breast cancer cohort: n = 10 patients, 25 samples; metastatic breast cancer cohort n = 8 patients, 27 samples) used. **a**–**i**) The box bounds the interquartile range divided by the median, with the whiskers extending to a maximum of 1.5 times the interquartile range beyond the box. Individual data points shown as dots. **a**, **c**, **e**–**i**) Wilcoxon rank sum tests, all *P* values two-sided.

B cell clones present at multiple time points during treatment (temporally persistent clones) or at multiple sites (immunosurveilling clones) were significantly enlarged compared with private clones, with BCR clone size correlating with both the number of time points and metastatic sites in which BCR clones were observed (Fig. 3a; *P* < 2.2 × 10^−^^16^, ordinal regression over the mean percentage clone size within each participant averaged over all sites observed), suggestive of immune surveillance by activated B cell clones. This directly shows that larger clones per site are associated with temporal persistence and immunosurveillance, rather than just a larger number of BCRs detected across all sites. Similarly, by classifying BCR clones as stem, clade or private depending on whether they were present in all, some or one tumor sample from the same participant, respectively, we observed that immunosurveilling and temporally persistent clones were significantly enlarged (stem > clade > private, *P* < 2.2 × 10^−16^, ordinal regression; Extended Data Fig. 4a).

Tumor-infiltrating BCRs were classified into four clone classes (A–D; Fig. 3a and Methods) based on whether they were (1) expanded or unexpanded within the tumor microenvironment, and (2) private to one site or shared between time points (temporally persistent) or multiple metastatic sites (immunosurveilling). There was no significant enrichment of BCR sequences with known binding to viral or bacterial antigens in these four clonal categories, indicating that these were not enriched for established systemic responses to non-cancer antigens and, therefore, did not just represent re-expansions of non-tumor-specific B cell clones (Extended Data Fig. 4b, Supplementary Table 3 and Methods). Expanded temporally persistent clones (clone class B) comprised the majority of tumor-infiltrating BCR sequences throughout the course of therapy in early breast cancer (Fig. 3b). Likewise, expanded immunosurveilling clones (clone class B) comprised the majority of tumor-infiltrating BCR sequences in metastatic disease (Fig. 3b). These clones were also present at higher proportions in liver and lung/pleura metastases compared to private expanded clones (class A), suggesting that they are highly activated in these sites. Interestingly, within lymph node metastases, there was no significant difference between class A and B clone proportions, suggesting that a large fraction of activated B cell clones in lymph nodes are resident and not undergoing immunosurveillance.

### Antigen experience of migratory and persistent clones

We next investigated whether the nature of shared B cell clones (clone classes B and D) was significantly distinct from private clones (clone classes A and C) based on BCR repertoire features. We calculated BCR CDR3 probability of generation (*P*_gen_) as a result of VDJ recombination (that is, the likelihood of being generated by chance rather than being individual specific) using OLGA^25^. We observed that clone class C (private unexpanded clones) had the highest probability of generation by chance, and the distribution was comparable to naive or antigen-inexperienced B cells from healthy peripheral blood mononuclear cells (Fig. 3c)^26^. The other clonal groups (B, C and D) had higher probabilities of BCR amino acid sequences resembling antigen-experienced BCRs, with the majority of these sequences being mutated and class switched, with clone class B (expanded and immunosurveilling) having the lowest *P*_gen_ scores. This suggests that the expanded immunosurveilling and temporally persistent clones are both selected on the basis of their BCR sequence and that these are likely to be participant-specific clones and from antigen-experienced B cells.

On encountering antigen, BCR sequences may diversify further via SHM, which introduces point mutations into the BCR, and class-switch recombination (CSR), which changes BCR isotype, to generate finely tuned humoral responses^10^. Measuring SHM and CSR between the different clone classes and by disease stage yielded three key observations. Firstly, expanded immunosurveilling clones (clone class B) had greater overall levels of class-switched BCRs (that is, lower levels of unswitched BCRs; Fig. 3d) compared to unexpanded private clones (clone class C). Furthermore, B cells infiltrating early tumors had lower levels of unswitched (IGHM/D) BCRs compared to metastasis-infiltrating B cells (Fig. 3d and Extended Data Fig. 4c). Secondly, the levels of SHM of tumor-infiltrating B cells varied by clone class (Extended Data Fig. 4d) and increased during treatment in early breast cancer, but this trend was reversed in the metastasis-infiltrating B cells (Fig. 3e and Extended Data Fig. 4e). These differences were driven by a higher proportion of low SHM BCRs and a lower proportion of high SHM BCRs in the metastasis-infiltrating B cells (Extended Data Fig. 4d). The association observed here of reduced SHM and CSR in metastasis-infiltrating B cells compared to B cells infiltrating the primary tumor site in early breast cancer is supported by the reduced expression levels of *AICDA*, which encodes a key enzyme associated with these processes (Extended Data Fig. 4f). Thirdly, the isotype usage proportions varied by clone class (Extended Data Fig. 4g) and varied with disease course, with IGHA1 increasing with time and IGHG1 decreasing with time (Fig. 3f), with this trend driven by clonal class B BCRs (Extended Data Fig. 4g). This is supported by the higher expression of IgA isotype switching and the lower expression of IgG isotype switching signatures in metastatic samples (Extended Data Fig. 4h).

Furthermore, tumors with high levels of both BCR SHM and class switching were associated with significantly higher levels of class B clonal B cells, as well as higher levels of B cell and T cell infiltration, TLS score, interferon gamma (IFN-γ) score and inflammation scores (Fig. 3g). The effect observed in the tumor was much more pronounced compared to that seen in healthy tissues (Extended Data Fig. 4i).

In summary, these data suggest that temporally persistent and immunosurveilling clones are significantly distinct from private clones by being clonally expanded and antigen experienced, rather than being naive B cells, in agreement with previous studies^11^. Higher levels of CSR and SHM are associated with higher levels of B cell and T cell infiltration and TLS scores, suggesting that the tumor microenvironment drives these differences.

### BCR centrality reveals sites of clonal diversification

**Fig. 4:**
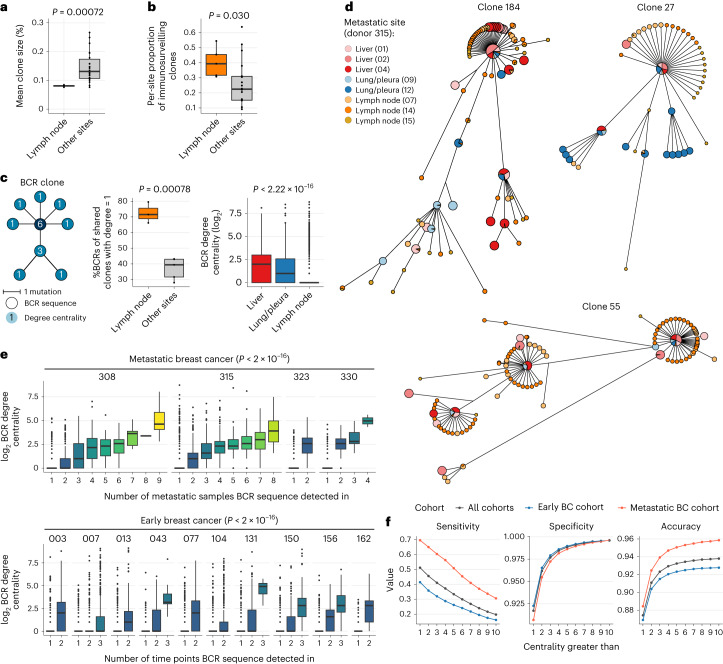
Higher BCR centrality describes clonal structure and predicts B cell immunosurveillance and persistence. **a**, Box plot showing mean BCR clone size in lymph nodes versus other sites (*n* = 5 lymph node, *n* = 22 other sites). **b**, Box plot showing per-site proportion of immunosurveilling clones in lymph nodes versus other sites (*n* = 5 lymph node, *n* = 22 other sites). **c**, Left, schematic of degree centrality as applied to BCR sequences within a BCR clone. Middle, box plots showing percentage of BCRs per sample with a degree centrality of 1 (that is, no progeny) in lymph nodes (*n* = 3) compared to other metastatic sites (*n* = 5) in participant 315. Right, box plots showing distribution of BCR degree centrality across different metastatic sites sampled in participant 315. **d**, BCR VDJ network plots showing three examples of expanded immunosurveillance clones shared between multiple metastatic sites in participant 315. These networks are based on maximum parsimony trees calculated from BCR sequence alignments. **e**, Box plots showing association between BCR degree centrality and the number of metastatic sites and therapy time points in which the BCR is observed. *P* values calculated using two-sided analysis of variance. **f**, Profile plots showing changes in sensitivity, specificity and accuracy at identifying immunosurveilling BCRs at different degree centrality thresholds in all samples, early breast cancer samples and metastatic breast cancer samples. **a**,**b**, Data from four participants with more than one metastatic site sampled (308, 315, 323 and 330) used. **a**–**c**, Wilcoxon rank-sum tests. All *P* values are two sided. **a**–**c**,**e**, The box bounds denote the interquartile range divided by the median, with the whiskers extending to a maximum of 1.5 times the interquartile range beyond the box. Individual data points are shown as dots.

**Extended Data Fig. 5:**
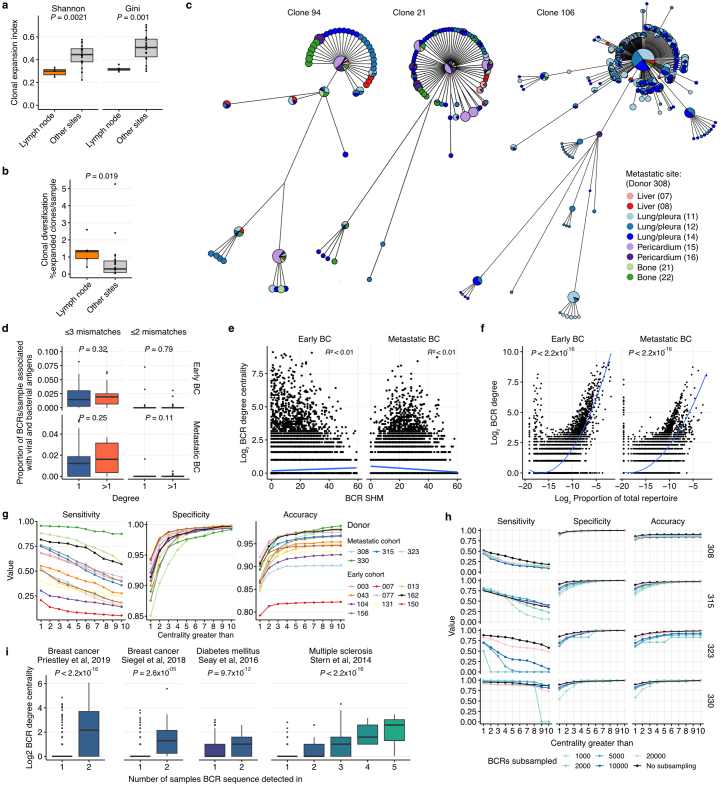
BCR degree centrality analyses. **a**) Boxplots showing clonal expansion as measured by the Shannon and Gini indices in lymph nodes versus other sites. **b**) Boxplots showing clonal diversity, as measured by the percentage of expanded BCR clones (≥4 unique VDJs) in lymph nodes versus other sites. **c**) BCR VDJ network plots showing three expanded immunosurveillance clones shared between multiple sites in patient 308. **d**) Boxplots showing proportion of unique BCRs/sample with CDR3 sequences matching a reference antibody database with known binding to viral or bacterial antigens with ≤3 and, ≤2 CDR3 mismatches, across BCRs with degree centrality = 1 and >1. **e**) Scatter plots showing lack of association between BCR degree centrality and BCR SHM in early and metastatic breast cancer cohorts. **f**) Scatter plots showing association between BCR degree centrality and proportion of total repertoire in early and metastatic breast cancer cohorts. Two-sided *P* value derived from polynomial regression. **g**) Profile plots showing changes in sensitivity, specificity, and accuracy at identifying immunosurveilling BCRs at different degree centrality thresholds in early and metastatic breast cancer cohorts. **h**) Profile plots showing changes in sensitivity, specificity and accuracy at identifying immunosurveilling BCRs at different centrality thresholds in four patients with metastatic breast cancer at five subsampling depths (1000, 2000, 5000, 10000 and 20000). **i**) Boxplots showing association between BCR degree centrality and the number of sites in which the BCR is observed in four external datasets (number of cases: n = 1 Priestley et al, n = 1 Siegel et al, n = 8 Seay et al, n = 3 Stern et al). Kruskal-Wallis tests, all *P* values two-sided. **a**,**b**) Data from four patients with more than one metastatic site sampled (308, 315, 323, 330) used (n = 5 lymph node, n = 22 other sites). **d**–**f**) Data from four patients with more than one metastatic site sampled (patients: 308, 315, 323, 330) and all patients (n = 10) with early breast cancer. **a**, **b**, **d**, **i**) The box bounds the interquartile range divided by the median, with the whiskers extending to a maximum of 1.5 times the interquartile range beyond the box. Individual data points shown as dots. **a**, **b**, **d**) Wilcoxon rank sum tests, all *P* values two-sided.

To determine whether B cell clonal diversification occurred within each metastasis or was localized to specific anatomical locations, per-sample BCR clonal expansion and diversification measures were calculated^26^. Lymph nodes had significantly lower levels of clonal unevenness, thus by extension, higher levels of clonal diversity (measured by the normalized mean clone size index (Fig. 4a) and Shannon and Gini indices (Extended Data Fig. 5a), *P* < 0.05 with effect sizes >1.33). However, there was a greater abundance of expanded clones in lymph node than non-lymph node sites (Extended Data Fig. 5b), indicating that there are more B cell clonal expansions in the lymph nodes, and only some clones are overrepresented in the non-lymph node sites. Additionally, lymph nodes had a higher proportion of unique BCRs from immunosurveilling clones compared to other sites (Fig. 4b). Together, this suggests that clonal diversification predominantly occurs within lymph nodes, and these are a main source of immunosurveilling B cells in metastatic breast cancer.

Next, we derived the BCR phylogenetic degree centrality, representing the number of edges connected to each BCR node in the network (Fig. 4c). This allowed us to distinguish between BCRs derived from B cells that underwent subsequent clonal diversification and were progenitors to many other BCR variants (high centrality) from those derived from B cells that did not undergo subsequent clonal diversification and were not progenitors to further BCR variants (unitary centrality). The majority of lymph node BCRs had a degree centrality of one compared to other metastatic sites (Fig. 4c), indicating that lymph nodes are key sites of clonal diversification where many exploratory variants are generated. Conversely, non-lymph node metastatic sites had a higher proportion of BCRs with degree centrality greater than one, indicating that these BCRs are predominantly variants of expanded clones under significant selection (that is, non-exploratory variants). These data suggest that higher levels of clonal diversification occur in lymph nodes, with high-centrality BCRs more likely to migrate to non-lymph node sites than low-centrality BCRs. There is minimal additional diversification in non-lymph node metastatic sites, and these typically do not undergo immunosurveillance to other sites.

### High BCR centrality of immunosurveilling and persistent BCRs

We next investigated B cell clonal relationships across sites to determine whether all members of expanded immunosurveilling clones (clone class B) underwent active metastatic immunosurveillance, or whether migration was restricted to a predictable subset of BCRs within each clone. BCRs from expanded clones (≥10 BCRs) were aligned and phylogenetic trees estimated to determine their lineage relationships. These were then represented as non-cyclic networks (Fig. 4c), with nodes representing unique BCRs and edges representing SHM between related BCRs. Visual representations of BCR clonal phylogenetic trees (Fig. 4d and Extended Data Fig. 5c) demonstrate this trend, with highly central BCRs shared between multiple sites and BCRs with a centrality of one typically observed in single sites.

Furthermore, BCR degree centrality was also strongly correlated with both (a) the number of metastatic sites in which the BCR was observed (*P* < 2.2 × 10^−16^; Fig. 4e), indicating that a small proportion of variants per activated clone, which are typically more central within the clone, perform immunosurveillance across multiple metastatic sites, and (b) the number of time points in which the BCR was observed (*P* < 2.2 × 10^−1^^6^; Fig. 4e), indicating that a small proportion of high-centrality BCR variants per activated clone are temporally persistent. This increased BCR degree centrality was not associated with systemic responses against noncancerous antigen (Extended Data Fig. 5d). BCR degree centrality was independent of BCR SHM level (Extended Data Fig. 5e), showing that immunosurveilling BCRs are not necessarily the most mutated versions of these clones, but rather represent local optima of the clonal response to its antigen. Finally, BCR degree centrality also correlated significantly with BCR frequency (Extended Data Fig. 5f; *P* < 2.2 × 10^−16^) in addition to immunosurveillance and clonal persistence. Together, this points to BCR clonal structure as a predictor of B cell activation, expansion and migratory potential.

We lastly determined whether BCR degree centrality would have sufficient power to predict immunosurveillance and clonal persistence. Indeed, degree centrality was highly predictive of BCR immunosurveilling status and clonal persistence, with a degree classification threshold greater than two resulting in an immunosurveilling and clonal persistence BCR identification accuracy greater than 80% (Fig. 4f and Extended Data Fig. 5g), which was robust to sequencing depth (Extended Data Fig. 5h).

The same association was observed in two independent breast cancer datasets^27,28^ (BCR data obtained following the deconvolution of bulk RNA-seq data; Extended Data Fig. 5i). We also observed that this trend of higher BCR centrality correlating with immunosurveillance is generalizable to noncancerous disease states, including autoimmunity (diabetes mellitus^29^ and multiple sclerosis^30^; Extended Data Fig. 5i).

In summary, BCRs diversify predominantly in the lymph nodes and only a small selection of B cells expressing these clonal BCR variants are able to perform immunosurveillance across other sites or are temporally persistent. Higher-centrality BCRs are more likely to be seen across a larger number of sites. These immunosurveillance and temporally persistent BCRs can be predicted from their centrality with respect to the overall clonal structure.

## Discussion

Anticancer immunosurveillance by B cells and T cells plays a central role in sculpting malignant clones, and disruption of this process is a hallmark of cancer^31^. A central finding of our study was that it appears that both arms of the adaptive immune response coevolve in a correlated fashion, suggesting common drivers of immune cell infiltration, selection and clonal expansion across metastatic sites. These adaptive immunity B cell and T cell clonal structures also correlate with the tumor mutational phylogenetic landscape, providing further support in favor of the immunoediting hypothesis^32^, where failure of the immune system to eliminate malignant cell populations results in a phase of equilibrium, in which the immune system limits but cannot eradicate the tumor, resulting in selection pressures that drive tumor evolution toward a state of reduced immunogenicity.

Mutated peptides can be presented on both MHC class I and class II molecules. MHC class II molecules are primarily expressed on professional antigen-presenting cells such as dendritic cells, B cells and macrophages, and predominantly present exogenously derived peptide antigens to CD4^+^T cells^33^. Indeed, B cells use a specialized MHC class II presentation to internalize and process BCR-bound antigen for presentation to CD4^+^T cells, which has been shown to influence the fate of both B and T cells^34,35^. The majority of intra-tumoral B cells have been shown to be non-antibody-secreting cells, but rather have a naive or memory phenotype with surface BCR^11,36,37^. The significant correlation between shared BCR sequences and MHC II, but not MHC I, supports the notion that B cells play a role in presenting antigen to T cells though BCR-dependent mechanisms^35^. Even though our data are unable to distinguish between CD4^+^ and CD8^+^ TCRs, they strongly support the hypothesis that tumor MHC class II neoantigens may be important in coordinating tumor-specific B and T cell responses, as the tumor MHC class II neoantigen landscape correlated with both B and T clonal structures. In keeping with this observation, MHC class II neoantigens have been recently shown to predict outcomes in HER2-negative breast cancer^38^ and associate with tumor-infiltrating lymphocytes and interferon signaling^39^.

The nature of B cells and T cells migrating between the tumor and draining lymph nodes is important for mounting effective antitumor immune responses, for TLS formation and for establishing long-term systemic memory, which are strongly associated with outcome^40^. However, despite the potential impact of B cells in antitumor responses and participant survival, the nature of B cell immunosurveillance across metastatic sites is unknown. Here we show that the majority of intra-tumoral B cells are temporally persistent and undergo tumor immunosurveillance across sites. These immunosurveilling and temporally persistent B cell clones are antigen experienced and isotype usages vary with disease stage. While some of these measures do not show a high correlation and causality remains unexplored, this is in line with previous studies showing a need for a diverse antibody repertoire for early neoplastic cell recognition and the critical role B cells play in anticancer immunity^41,42^.

Finally, we show that not all BCRs from expanded shared clones perform immunosurveillance. We have generated a pipeline that uses network graph theory to predict which BCR sequences within an immunosurveilling BCR clone perform cross-site immunosurveillance. These B cells tend to have higher BCR degree centrality but do not have the highest level of SHM within the clone. Therefore, these are likely to represent local optima of the B cell clonal response to its antigen. Furthermore, we show that BCR degree centrality can be used to predict BCR clonal persistence and demonstrate its generalizability across other breast cancer datasets and non-cancer datasets. While the concept of BCR degree centrality has been used to describe B cell population distributions^43^, we show functional differences between low-centrality and high-centrality B cell clonal variants for the prioritization of specific BCRs. Indeed, this study shows functional and BCR-dependent associations with B cell immunosurveillance and clonal persistence. While these findings are primarily observational, hence the significance in the broader context of cancer immune response and participant outcomes is mostly correlative, they potentially lay the foundation for expediting the discovery of tumor-specific or persistent B cell clones. Given these findings, we hypothesize that this can be used to develop personalized antibody-based therapies based on BCR network degree centrality.

## Methods

### Study population

Eight participants with metastatic breast cancer enrolled within the Vall d’Hebron Institute of Oncology (VHIO) Warm Autopsy Program were included within this study. Ethical approval from the institutional review board of the Vall d’Hebron University Hospital (Barcelona, Spain) was obtained for the use of biospecimens with linked pseudo-anonymized clinical data. The ten participants with primary invasive early breast cancer included in this study were enrolled in the TransNEO study at Cambridge University Hospitals NHS Foundation Trust. Appropriate ethical approval from the institutional review board (research ethics ref.12/EE/0484) was obtained for the use of biospecimens with linked pseudo-anonymized clinical data. All participants provided informed consent for sample collection, and all participants consented to the publication of research results. Full details regarding sample collection, DNA and RNA extraction, library preparation and sequencing have been published elsewhere^8,16^. No statistical methods were used to predetermine sample sizes, but our sample sizes are similar to those reported in previous publications^16^. When performing statistical testing, we assessed whether the data met the assumptions of the tests used.

### DNA somatic mutation calling and neoantigen prediction

Somatic mutations (Fig. 2d) and predicted HLA class I neoantigens (Fig. 2f) were identified from whole-exome sequencing data and tumor phylogenetic trees (Fig. 2e) were generated using OncoNEM^44^, as previously described^16^. MHC class II allele genotyping was performed on the normal tissue DNA sequencing data using HLA-HD^45^ (version 1.4) using default parameters. MHC class II neoantigens were predicted from the whole-exome mutation data using mixMHC2pred^46^ (version 1.2) and putative candidates with a percentage rank cutoff of 2% were retained (Fig. 2f).

### TME composition and activity deconvolution from bulk RNA-seq

RNA-seq data from the early and metastatic breast cancer cohorts were processed as previously described^8,16^. Briefly, FASTQ files were aligned to the GRCh37 assembly of the human genome using STAR^47^ (version 2.5.2b) in two-pass mode and counting of reads aligned over exonic features performed using HTSeq^48^ (version 0.6.1p1) in read strand-aware union overlap resolution mode.

Immune cell enrichment was performed using MCPcounter^22^ (version 1.2.0), using as input normalized log-transformed RNA-seq expression data (Extended Data Fig. 2b), and enrichment over 14 cell types using 60 genes^21^ (Figs. 2c and 3g and Extended Data Figs. 2c,d and 4i). In Extended Data Fig. 2e, published TCGA B cell and T cell enrichment scores are shown^21^. Correlations between tumor microenvironment components shown in Fig. 2c and Extended Data Fig. 2b were generated using the cor function in the base R stats package and visualized using the corrplot package (version 0.92). The TLS gene signature (*CCL19*, *CCL21*, *CXCL13*, *CCR7*, *CXCR5*, *SELL*, *LAMP3)*^23^ shown in Fig. 3g and Extended Data Figs. 2d,e and 4i was calculated using gene-set enrichment analysis. TCGA TLS enrichment scores (Extended Data Fig. 2e) were obtained using FPKM normalized counts provided by TCGA (Genomic Data Commons data release 37.0).

The cytolytic activity score^20^ (CYT; Extended Data Fig. 2c) was computed as the geometric mean of *GZMA* and *PRF1* expression (TPM, 0.01 offset). The T cell inflamed score^49^ (Fig. 3g and Extended Data Figs. 2c and 4i) was computed using the GSVA^50^ R package (version 1.38.2) using as input the log-normalized expression of 18 inflammatory genes (*TIGIT*, *CD27*, *CD8A*, *PDCD1LG2*, *LAG3*, *CD274*, *CXCR6*, *CMKLR1*, *NKG7*, *CCL5*, *PSMB10*, *IDO1*, *CXCL9*, *HLA-DQA1*, *CD276*, *STAT1*, *HLA-DRB1* and *HLA-E*), while the interferon-γ score^49^ (Fig. 3g and Extended Data Figs. 2c and 4i) was computed using gene-set variation analysis of six genes (*IFNG*, *STAT1*, *IDO1*, *CXCL10*, *CXCL9* and *HLA-DRA*). The B cell activation score shown in Extended Data Fig. 2c was computed using GSVA on the MSigDB^51^ (version 7.3) C5 Gene Ontology Biological Processes POSITIVE_REGULATION_OF_B_CELL_ACTIVATION (GO:0050871) gene set, using as input the log_2_ TPM expression, with 0.01 offset.

### Healthy tissue GTEx isotype analysis

In the healthy tissue BCR isotype analysis shown in Extended Data Fig. 1c, normalized gene counts (TPM) were downloaded from the GTEx^17^ consortium website (version 8, https://gtexportal.org/home/datasets) and the expression of IGH isotypes retained. Expression data were available for 3,905 samples from organ sites sampled within this study (GTEx *n*: brain = 2,642, breast = 459, liver = 226, lung = 578). In Extended Data Fig. 1c, the heat map shows the proportion of isotype TPM expression per organ site. In Extended Data Fig. 1d, the median *z*-score scaled expression of BCR isotypes is shown. The expression values of *CD3D*, *CD3G*, *CD3E* and *CD247*, which encode for the four different parts of the CD3 complex, were summed to calculate TCR expression. In Extended Data Fig. 4i, samples with high expression of unswitched transcripts were defined as those with a >50th percentile expression of IGHD/IGHM genes, while those with low expression of unswitched transcripts were defined as those with a ≤50th percentile expression of IGHD/IGHM.

### BCR library preparation and sequencing

BCR libraries were prepared from RNA samples extracted from 27 metastatic sites and 25 primary breast tumors. BCR variable heavy domains were first amplified using a protocol we have previously described^52^. Briefly, RNA was reverse transcribed to cDNA using a mixture of IgA/IgD/IgE/IgG/IgM isotype specific primers, incorporating 15 nucleotide unique molecular identifiers (UMIs). The resulting cDNA was used as a template for PCR amplification using a set of six FR1-specific forward primers including sample-specific barcode sequences (seven nucleotides) along with a reverse primer specific to the reverse transcription primer. For three of the replicate libraries, a modified primer set was used where the sample-specific barcode was instead incorporated into the reverse transcription primers after the UMI.

BCR variable heavy domain amplicons (~450 bp) were quantified by TapeStation (Beckman Coulter) and subjected to gel purification. Dual-indexed sequencing adapters (KAPA) were ligated onto ≤500 ng of amplicon per sample using the HyperPrep library construction kit (KAPA). The adaptor-ligated libraries were finally PCR amplified (initial denaturation at 95 °C for 1 min, for 2–83 cycles at 98 °C for 15 s, 60 °C for 30 s, 72 °C for 30 s and a final extension at 72 °C for 1 min). The libraries were sequenced on an Illumina MiSeq using the 2 × 300-bp chemistry.

### BCR-sequencing processing

Raw BCR-sequencing reads were processed for analysis using the Immcantation framework, using previously described parameters (docker container v3.0.0)^52,53^. Briefly, paired-end reads were joined based on a minimum overlap of 20 nucleotides, and a maximum error of 0.2, and reads with a mean Phred score below 20 were removed. Primer regions, including UMIs and sample barcodes, were then identified within each read, and trimmed. Together, the sample barcode, UMI, and constant region primer were used to assign molecular groupings for each read. Within each grouping, usearch^54^ was used to subdivide the grouping, with a cutoff of 80% nucleotide identity, to account for randomly overlapping UMIs. Each of the resulting groupings is assumed to represent reads arising from a single RNA. Reads within each grouping were then aligned, and a consensus sequence determined. To remove low-level noise, molecular groupings with two or fewer sequences contributing to the UMI consensus were filtered out (Supplementary Table 1). Duplicate reads were then collapsed into a single processed sequence. IgBlast^55^ (version 1.14.0) was used to annotate the processed sequences, and unproductive sequences were removed. Sequence data from replicate libraries were then pooled for analysis.

### BCR clonotype assembly

Annotation of TCR and BCR sequences were performed using IMGT/HighV-QUEST^56^ (version 1.8.5) and clonotype assembly performed using MRDARCY^57^, which was run using default parameters. B cell clones are groups of B cells from an individual that derive from the same pre-B cell, and thus have identical BCR sequences or BCR sequences related by SHM. Computationally, BCRs from clonal B cells can be clustered together via network generation using a previously described pipeline^26^. Briefly, each vertex represents a unique sequence, and the relative vertex size is proportional to the number of identical reads. Edges join vertices that differ by single-nucleotide non-indel differences and clusters are collections of related, connected vertices. A clone (cluster) refers to a group of clonally related B cells, each containing BCRs with identical CDR3 regions and IGHV gene use, or differing by single point mutations, such as through SHM. Likewise, a T cell clone (cluster) refers to a group of related T cells arising from the same pre-T cell, each containing TCRs with identical CDR3 regions and TCRV gene usage.

### BCR CDR3 overlap with reference pathogen antibody libraries

A reference antibody database with known binding to viral or bacterial antigen was constructed from existing public databases: the structural antibody database^58^, abYsis human antibody database^59^ and the immune epitope database^60^. Antibody sequences corresponding to synthetic fusion proteins and animal-derived BCRs were excluded.

After preprocessing, 5,800 antibody sequences reacting to antigens were retained, including those derived from human immunodeficiency virus-1 (*n* = 3,525), *Clostridium tetani* (*n* = 817), influenza A (*n* = 486), vaccinia virus (*n* = 92), hepatitis C virus (*n* = 80), *Streptococcus pneumoniae* (*n* = 59), *Staphylococcus aureus* (*n* = 38) and human betaherpesvirus 5 (*n* = 32) were used for downstream analysis (Supplementary Table 2).

To determine potential matches, we screened the cancer CDR3 amino acid sequences to the reference antibody database, allowing for up to three amino acid mismatches by fuzzy string matching via a custom Python script. The proportions of BCRs/sample associated with known binding to viral or bacterial antigen across clone classes (Extended Data Fig. 4b) and degree centrality (Extended Data Fig. 5d) were calculated to show that the observations made were not secondary to established systemic responses to non-cancer antigens.

### TCR library preparation and sequencing

TCR-sequencing library preparation, sequencing and repertoire identification and network analysis performed by us have been described previously^16^. Briefly, MiSeq libraries were prepared using the same protocol as for the BCR libraries. Raw MiSeq reads were filtered for base quality, primer and constant region trimming, annotation and clustering using the same protocol as for the BCR libraries but using TCR as the chain parameter.

### Clonal overlap between metastatic sites

In Fig. 2 and Extended Data Fig. 2, the clonal repertoire analyses for participants 308 and 315 that were dependent on sequencing depth were generated by subsampling each sample to 90% of the number of unique VDJ sequences present in the sample with the lowest depth (unique VDJ subsampling thresholds: participant 308: *n* = 980 (BCR), 4,657 (TCRα), 2,620 (TCRβ); participant 315: *n* = 1,524 (BCR), 3,199 (TCRα), 2,535 (TCRβ). Throughout the paper, we have used the term ‘relative level’ to indicate that the analyses were performed using subsampled data.

In Fig. 2b,d,f and Extended Data Fig. 2a,f,g, the relative level of shared BCR/TCR VDJ sequences was computed by calculating the number of shared VDJ sequences between different metastatic sites in 10,000 subsampling operations and then computing the median of the number of overlaps across iterations. In Fig. 2e, the median Jaccard coefficient of shared VDJ sequences derived in the same 10,000 subsampling operations was used to generate BCR and TCR similarity matrices, from which hierarchical clustering was performed to generate the BCR and TCR clonal similarity trees via the hclust function in R using the ward.D2 agglomeration method. In the spatio-migratory maps of B cell clonal migration shown in Extended Data Fig. 2f,g, the clonal repertoire analyses for participants 308 and 315 were generated by calculating the median number of shared BCR clones across the same 10,000 subsampling operations.

### Clonal overlap correlations with tumor genomic landscape

The tumor phylogenetic trees were generated using OncoNEM^44^, as previously described by us^16^. The hclust (hierarchical clustering) function in the base R stats package was used to compute the BCR, TCR and genomic trees using the ward.D2 agglomeration method. The comparison of the hclust objects was done using the cophenetic correlation, using the cor_cophenetic function from the dendextend package (version 1.15.2)^61^. A permutation test was used to calculate correlation one-sided *P* values, where the tree labels were randomly shuffled for 100 permutations, while keeping the tree topologies constant. The comparison of the BCR and TCR Jaccard clustering trees with the genetic trees was done by using the cophenetic definition for edge-weighted trees. In this version of the cophenetic, the distance between each pair of nodes is the sum of the weights of edges along the path connecting these pairs of nodes.

### BCR and TCR clonotype classification

In all participants with more than one tumor sampled (metastatic breast cancer cohort participants: 308, 315, 323, 330; early breast cancer cohort: all participants; Fig. 1), the clone proportion per sample was calculated by dividing the number of UMIs from each clone identified using MRDARCY with the total number of UMIs present in the sample. BCR clones were classified as stem, clade or private depending on whether they were observed in all, some or a single sample from the same participant, respectively (Extended Data Fig. 4a). Stem and clade clones were considered to be immunosurveilling given that they were present in more than one metastatic sample from a single participant.

We further refined the stem, clade and private clone classification by taking into account clone size (percentage of UMIs) to identify clonal expansion. We fitted a Gaussian mixture model to the log percentage UMI values of all BCR sequences of all early and metastatic breast cancer samples using the MClust (version 5.4.9)^62^ R package to identify an overall BCR clone size cutoff threshold for expanded versus unexpanded clones. This threshold was set to ensure representation of all four clonal classes in all samples and that the expanded clones represented less than 10% of the total repertoire. Using this threshold, BCR clones were classified into four categories: (A) private and expanded, (B) shared and expanded, (C) private and unexpanded and (D) shared and unexpanded (Fig. 3a). Clones where clone size was above the cutoff threshold in some sites (that is, expanded) and below the threshold in others (that is, unexpanded) were classified as expanded.

### CDR3 probability of generation analysis

We calculated BCR CDR3 *P*_gen_ as a result of VDJ recombination with OLGA^25^ version 1.2.4 using as input the default human B cell heavy chain model and the amino acid CDR3 sequence of each BCR (Fig. 3c). In Fig. 3c, *P*_gen_ scores derived from BCR-sequencing data obtained from the peripheral blood mononuclear cells from a published healthy participant^26^ are shown. Antigen-experienced BCRs were defined as those that were class switched (IgA, IgE, IgG) and had more than four somatic mutations. Antigen-inexperienced BCRs were defined as non-class-switched BCRs (IgD and IgM) with four or fewer mutations.

### Isotype usages and SHM across BCR clone classes

In Fig. 3d, the number of UMIs in each clone per IGH isotype were counted for each sample and summarized by summing the UMI counts by clone class (A, B, C, D) for each isotype/sample, resulting in 192 sample/clone class combinations (48 samples × 4 BCR clone classes) for all 9 BCR isotypes. The total proportion of unswitched BCRs comprised the sum of the proportion of IgD and IgM UMI BCRs. In Fig. 3f, the total proportion of each IGH isotype across sequential samples obtained during therapy in early breast cancer and metastatic samples is shown. Statistical comparisons between early breast cancer time points were performed using an ordinal logistic regression to identify whether there was a monotonic association between IGH isotype proportion and time point. Statistical comparisons between early and metastatic breast cancer samples were performed using Wilcoxon rank-sum tests. In Extended Data Fig. 4g, the data plotted in Fig. 3f are subset across the four BCR clone classes.

BCRs were classified into four SHM categories (no, low, high and very high SHM) using the normalmixEM function from the mixtools R package (version 2.0.0), providing as input the log SHM count. The thresholds used were 0–1 mutation, 1–10 mutations, 11–33 mutations and >33 mutations for the no, low, high and very high SHM categories, respectively. In Extended Data Fig. 4d, the proportion of BCRs for each of the four SHM classes per sample is shown across the four BCR clone classes. In Fig. 3e, highly mutated BCRs were defined as those BCRs classified as having high and very high SHM counts. Statistical comparisons between early breast cancer time points were performed using an ordinal logistic regression to identify whether there was a monotonic association between the percentage of highly mutated BCRs and time point. Statistical comparisons between early and metastatic breast cancer samples were performed using Wilcoxon rank-sum tests.

In the analyses shown in Fig. 3g, all samples from all participants were used. The sample isotype usage was calculated by summing the total number of BCR UMIs per isotype per sample and then dividing this by the total number of UMIs within the sample, as described previously. The total proportion of unswitched BCR comprised the sum of the proportion of IgD and IgM BCRs. The mean sample BCR mutation count was calculated by first calculating the mean SHM per clone per sample, and then calculating the mean SHM per sample (so that larger clones are not overrepresented). Samples with high SHM and CSR were defined as those with a >50th percentile SHM and CSR, while those with low SHM and CSR were defined as those with a ≤50th percentile SHM and CSR (Fig. 3g). In Fig. 3g, data from participants with more than one tumor site sampled are shown (early breast cancer cohort: all participants (*n* = 10), metastatic breast cancer cohort participants: 308, 315, 323, 330), as classification into the four clonal groups required the sampling of more than one site/participant. In Fig. 3g, all samples from all participants are shown.

### BCR clonal expansion and diversification

We calculated BCR clonal expansion by first subsampling each tumor’s BCR-sequencing data to 90% of the number of unique UMIs present in the sample with the lowest depth and summing the total number of UMIs associated with each unique BCR VDJ sequence. The Gini, Shannon index and mean clone sizes were calculated using the ineq R package (version 0.2–13), the posterior R package (version 1.4.1) and custom code, respectively. The mean of 1,000 iterations was used to calculate the final clonal expansion metrics (Fig. 4a and Extended Data Fig. 5a).

To calculate the per-site proportion of immunosurveilling clones (Fig. 4b), the total number of unique VDJ sequences per clone across all samples was calculated, and clones that were present in more than one site and had at least four unique VDJs in at least one metastatic site retained. The proportion of each of these clones across all samples was then calculated by dividing the total number of VDJs per clone per sample by the sum of the number of VDJs for that clone in all samples. The mean of these clone proportions per site was then calculated (Fig. 4b). In Extended Data Fig. 5b, the percentage of clones per sample that had at least four unique VDJ sequences were calculated.

### BCR clonal network analysis

Network clustering of BCR clones was performed using MRDARCY^57^ in participants with more than one site sampled (metastatic breast cancer cohort: participants 308, 315, 323 and 330, early breast cancer cohort: all 10 participants). BCRs were clustered using a sequence identity threshold of 0.95, and clones that were present in a minimum of two tumor samples for each participant and had a minimum of ten unique BCR sequences were retained (number of clones retained in metastatic dataset: participant 308 = 204; participant 315 = 733; participant 323 = 85; participant 330 = 23).

For each BCR clone, the ends of the multiple sequence alignment were trimmed until 95% of all BCR sequences had an aligned nucleotide at the end of the sequence, with a minimum trimmed length of 80 nucleotides required for network clustering to be performed. A distance matrix was subsequently constructed for all sequences per clone, identical BCR sequences grouped together into clusters, and the abundance of these clusters across metastatic sites was calculated by dividing the total number of UMIs present in the cluster by the total number of UMIs in the sample being analyzed. BCR clone network diagrams were generated by computing the pairwise Hamming distances between sequences using the phangorn^63^ R package (version 2.7.1), followed by neighbor-joining tree estimation and phylogenetic tree construction and optimization using the pml and optim.pml functions in phangorn (Fig. 4c,d and Extended Data Fig. 5c).

To calculate the degree of a BCR sequence, a minimum spanning tree was calculated on the Hamming distance matrix using the mst function in the ape^64^ R package (version 5.6), which was then converted into an undirected graph using the graph_from_adjacency_matrix function in the igraph R package (version 1.2.10). The degree centrality was then computed using the degree function in igraph (Fig. 4c–f and Extended Data Figs. 5c–h).

We validated in our network clustering findings in four independent datasets (Extended Data Fig. 5i). Two metastatic breast cancer datasets (from the Hartwig Medical Foundation (HMF)^27^ and the Rapid Autopsy tumor Donation program (RAP) at the UNC at Chapel Hill^28^) were identified and TRUST4 (ref. ^65^) was used to reconstruct the BCR immune receptor repertoires from the RNA-seq data, which were then processed using MRDARCY. Sixteen participants in the HMF dataset had breast tumor RNA-seq data for more than one metastatic deposit and clonotype assembly, and intra-participant comparison was only possible in one participant (participant ID: HMFN_0320), which had a higher coverage (1,085 BCRs identified in one sample and 1,757 in another). Similarly, clonotype assembly and intra-participant comparison were possible in one participant in the RAP dataset (participant ID: 828433). BCR-sequencing data for diabetes^29^ and a multiple sclerosis^30^ datasets were downloaded from the iReceptor gateway^66^ and processed using MRDARCY. Eight participants in the diabetes dataset and three participants in the multiple sclerosis dataset had multisite BCR-sequencing data for which clonotype assembly and intra-participant comparisons were possible.

We have created and uploaded an R framework hosted at https://github.com/sjslab/BCR-Immunosurveillance to generate network clustering of BCR clones and compute the centrality analyses from BCR repertoire data derived from BCR sequencing, as well as BCR repertoire data obtained from bulk RNA-seq data.

To determine the predictability of immunosurveilling clones based on BCR degree in the early and metastatic breast cancer cohorts (Fig. 4f and Extended Data Fig. 5g,h), we calculated the sensitivity, specificity and accuracy of a classification that categorizes BCRs as immunosurveilling or not based on a series of degree cutoffs (>1, >2 >10). Model performance metrics were generated using the confusionMatrix function in the caret (version 6.0-90) R package.

### Reporting summary

Further information on research design is available in the Nature Portfolio Reporting Summary linked to this article.

## Online content

Any methods, additional references, Nature Portfolio reporting summaries, source data, extended data, supplementary information, acknowledgements, peer review information; details of author contributions and competing interests; and statements of data and code availability are available at 10.1038/s41590-024-01821-0.

### Supplementary information

Reporting Summary

Supplementary Tables 1–3 Supplementary Table 1: Clinical information for the metastatic breast cancer cohort and analyzed samples. Supplementary Table 2: Clinical information for the early breast cancer cohort and analyzed samples. Supplementary Table 3: Reference antibody database with known binding to viral or bacterial antigen.

## Data Availability

Sequence data (aligned to the GRCh37 of the human genome) have been deposited in the European Genome-phenome Archive (EGA), which is hosted by the EBI and the CRG, under the accession codes EGAS00001002703 (tumor DNA and RNA) and https://ega-archive.org/studies/EGAS00001006976 and https://ega-archive.org/studies/EGAS50000000241 (BCR-sequencing data). Example processed data are available at https://github.com/sjslab/BCR-Immunosurveillance/.

## Code availability

Our R framework for the network clustering and centrality analyses of BCR repertoire data derived from BCR-sequencing or bulk RNA-seq data is made available to accelerate the identification of potential immunosurveilling and clonally persistent antibodies (https://github.com/sjslab/BCR-Immunosurveillance). The R source code used to run the analyses and generate the figures shown in this paper is also available at this repository.
