# Supplementary material for: Predictability of B cell clonal persistence and immunosurveillance in breast cancer
Source: Nat Immunol. 2024 May 2;25(5):916–24. doi: 10.1038/s41590-024-01821-0 (PMC11065701; doi:10.1038/s41590-024-01821-0)
Supplement: Supplementary file 1 — Reporting Summary [file 41590_2024_1821_MOESM1_ESM.pdf]

nature portfolio

Corresponding author(s): Stephen John Sammut, Carlos Caldas, Rachael Bashford-Rogers

Last updated by author(s): Mar 12, 2024

Reporting Summary

Nature Portfolio wishes to improve the reproducibility of the work that we publish. This form provides structure for consistency and transparency in reporting. For further information on Nature Portfolio policies, see our Editorial Policies and the Editorial Policy Checklist.

Statistics

For all statistical analyses, confirm that the following items are present in the figure legend, table legend, main text, or Methods section.

n/a	Confirmed	
☐	☒	The exact sample size (*n*) for each experimental group/condition, given as a discrete number and unit of measurement
☐	☒	A statement on whether measurements were taken from distinct samples or whether the same sample was measured repeatedly
☐	☒	The statistical test(s) used AND whether they are one- or two-sided<br>*Only common tests should be described solely by name; describe more complex techniques in the Methods section.*
☐	☒	A description of all covariates tested
☐	☒	A description of any assumptions or corrections, such as tests of normality and adjustment for multiple comparisons
☐	☒	A full description of the statistical parameters including central tendency (e.g. means) or other basic estimates (e.g. regression coefficient) AND variation (e.g. standard deviation) or associated estimates of uncertainty (e.g. confidence intervals)
☐	☒	For null hypothesis testing, the test statistic (e.g. *F*, *t*, *r*) with confidence intervals, effect sizes, degrees of freedom and *P* value noted<br>*Give P values as exact values whenever suitable.*
☒	☐	For Bayesian analysis, information on the choice of priors and Markov chain Monte Carlo settings
☒	☐	For hierarchical and complex designs, identification of the appropriate level for tests and full reporting of outcomes
☐	☒	Estimates of effect sizes (e.g. Cohen's *d*, Pearson's *r*), indicating how they were calculated

Our web collection on statistics for biologists contains articles on many of the points above.

Software and code

Policy information about availability of computer code

Data collection

Clinical data was collected in Microsoft Excel (as part of the Office 365 suite).

Data analysis

List of software used:
• HLA-HD: version 1.4
• HTSeq: version 0.6.1p1
• IgBlast: version 1.14.0
• IMGT/HighV-QUEST: version 1.8.5
• Immcantation framework: docker container v3.0.0
• mixMHC2pred: version 1.2
• MRDARCY https://github.com/Bashford-Rogers-lab/MRDARCY
• OLGA: version 1.2.4 https://github.com/statbiophys/OLGA
• OncoNEM https://bitbucket.org/edith_ross/onconem/src/master/
• STAR: version 2.5.2b
• TRUST4: version 1.0.11

R version 4.1.2 and associated packages:
• ape: version 5.6
• caret: version 6.0-90
• corrplot: version 0.92
• dendextend: version 1.15.2

• GSVA: version 1.38.2
• igraph: version 1.2.10
• MClust: version 5.4.9
• MCPcounter: version 1.2.0
• phangorn: version 2.7.1

Python version 3.10.1

The code to recreate analyses described in this manuscript has been uploaded to https://github.com/sjslab/BCR-Immunosurveillance

For manuscripts utilizing custom algorithms or software that are central to the research but not yet described in published literature, software must be made available to editors and reviewers. We strongly encourage code deposition in a community repository (e.g. GitHub). See the Nature Portfolio guidelines for submitting code & software for further information.

Data

Policy information about availability of data

All manuscripts must include a data availability statement. This statement should provide the following information, where applicable:

- Accession codes, unique identifiers, or web links for publicly available datasets
- A description of any restrictions on data availability
- For clinical datasets or third party data, please ensure that the statement adheres to our policy

Sequence data (aligned to the GRCh37 of the human genome) have been deposited at the European Genome-phenome Archive (EGA), which is hosted by the EBI and the CRG, under accession number EGAS00001002703 (Tumour DNA and RNA) and EGA00002343328 (BCR sequencing data). Once approval from the data access committee is secured processed data will be provided through direct communication with corresponding authors. Example processed data is available at https://github.com/sjslab/BCR-Immunosurveillance .

Human research participants

Policy information about studies involving human research participants and Sex and Gender in Research.

Reporting on sex and gender	Eight women with metastatic breast cancer and ten women with early breast cancer undergoing neoadjuvant therapy were analysed in this study.
Population characteristics	Eight women analysed in this study had lethal metastatic breast cancer: their detailed clinical information, including treatments, have been previously published: https://doi.org/10.1016/j.celrep.2019.04.098<br><br>Ten women analysed in this study had early breast cancer and were treated with preoperative therapies: their detailed clinical information, including treatments, have been previously published: https://doi.org/10.1038/s41586-021-04278-5
Recruitment	Eight patients with metastatic breast cancer who underwent post-mortem warm autopsies were included in this study. All patients were enrolled as part of the Vall d'Hebron Institute of Oncology (VHIO) Warm Autopsy Program. All ten women with early breast cancer were enrolled to the TransNEO study at Cambridge University Hospitals NHS Foundation Trust.
Ethics oversight	Metastatic breast cancer cohort: research autopsies were performed under VHIO Warm Autopsy Program protocols approved by the institutional review board (IRB) of Vall d'Hebron University Hospital (Barcelona, Spain).<br><br>Early breast cancer cohort: East of England Research Ethics Committee: 12/EE/0484.

Note that full information on the approval of the study protocol must also be provided in the manuscript.

Field-specific reporting

Please select the one below that is the best fit for your research. If you are not sure, read the appropriate sections before making your selection.

☒ Life sciences ☐ Behavioural & social sciences ☐ Ecological, evolutionary & environmental sciences

For a reference copy of the document with all sections, see nature.com/documents/nr-reporting-summary-flat.pdf

Life sciences study design

All studies must disclose on these points even when the disclosure is negative.

Sample size	8 women with lethal metastatic breast cancer and 10 women with early breast cancer were recruited to this study. No statistical methods were used to pre-determine sample sizes but our sample sizes are similar to those reported in previous publications.
Data exclusions	No data was excluded. BCR sequencing was performed in cases which had remaining RNA extracted.
Replication	For two of the metastatic tumour samples, two replicate BCR libraries were created, and for three of the metastatic samples, three replicate BCR libraries were created. High levels of BCR VDJ sharing were observed in technical replicates. All attempts at replication were successful as shown in Extended Data Figure 1e.
Randomization	Randomization not applicable - all cases were treated with standard of care therapy regimens.
Blinding	Blinding not applicable - no group allocations.

Reporting for specific materials, systems and methods

We require information from authors about some types of materials, experimental systems and methods used in many studies. Here, indicate whether each material, system or method listed is relevant to your study. If you are not sure if a list item applies to your research, read the appropriate section before selecting a response.

Materials & experimental systems

n/a	Involved in the study	
☒	☐	Antibodies
☒	☐	Eukaryotic cell lines
☒	☐	Palaeontology and archaeology
☒	☐	Animals and other organisms
☒	☐	Clinical data
☒	☐	Dual use research of concern

Methods

n/a	Involved in the study	
☒	☐	ChIP-seq
☒	☐	Flow cytometry
☒	☐	MRI-based neuroimaging
